# Core values of patients with advanced cancer considering participation in an early-phase clinical trial: a qualitative study

**DOI:** 10.1007/s00520-022-07200-5

**Published:** 2022-06-08

**Authors:** Jelle L. P. van Gurp, Liza G. G. van Lent, Nicole Stoel, Carin C. D. van der Rijt, Maja J. A. de Jonge, Saskia M. Pulleman, Julia C. M. van Weert, Jeroen Hasselaar

**Affiliations:** 1 Department of IQ Healthcare, Radboud University Nijmegen Medical Center, P.O. Box 9101, 6500 HB Nijmegen, the Netherlands; 2 Department of Medical Oncology, Erasmus MC Cancer Institute, Rotterdam, the Netherlands; 3 Department of Anaesthesiology, Pain & Palliative Medicine, Radboud University Medical Centre, Nijmegen, the Netherlands; 4 Department of Medical Oncology and Clinical Pharmacology, Antoni Van Leeuwenhoek, the Netherlands Cancer Institute, Amsterdam, the Netherlands; 5 Department of Communication Science, Amsterdam School of Communication Research (ASCoR) and University of Amsterdam, Amsterdam, the Netherlands

**Keywords:** Cancer, Oncology, Phase 1 clinical trial, Patient preference, Quality of life, Shared decision-making, Communication, Clinical ethics

## Abstract

**Objective:**

This article identifies the core values that play a role in patients’ decision-making process about participation in early-phase clinical cancer trials.

**Methods:**

Face-to-face, semi-structured serial interviews (*n* = 22) were performed with thirteen patients with advanced cancer recruited in two Dutch specialized cancer centers. In a cyclic qualitative analysis process, open and axial coding of the interviews finally led to an overview of the values that are woven into patients’ common language about cancer and clinical trials.

**Results:**

Six core values were described, namely, acceptance creates room for reconsideration of values, reconciliation with one’s fate, hope, autonomy, body preservation, and altruism. Previously found values in advanced cancer, such as acceptance, hope, autonomy, and altruism, were further qualified. Reconciliation with one’s fate and body preservation were highlighted as new insights for early-phase clinical cancer trial literature.

**Conclusions:**

This article furthers the understanding of core values that play a role in the lives and decision-making of patients with advanced cancer who explore participation in early-phase clinical cancer trials. These values do not necessarily have to be compatible with one another, making tragic choices necessary. Understanding the role of core values can contribute to professional sensitivity regarding what motivates patients’ emotions, thoughts, and decisions and help patients reflect on and give words to their values and preferences. It supports mutual understanding and dialog from which patients can make decisions according to their perspectives on a good life for themselves and their fellows in the context of participation in an early-phase clinical cancer trial.

**Supplementary Information:**

The online version contains supplementary material available at 10.1007/s00520-022-07200-5.

## Background

Many adult patients with advanced cancer will reach a moment when standard systemic or anticancer therapy is not or no longer available. If they are still in relatively good health, it can be suggested that they participate in early-phase clinical trials. Compared to other patient populations facing the opportunity to participate in a trial, patients with advanced cancer are likely to be torn by both a reluctance to (due to a combination of a history of medicalization, often satisfying relationships with “their” health care professionals, and a lack of feeling in control) and an enthusiasm for trial participation (due to the immediate threat to life and/or the threat of pain and other severe symptoms) [38]. Clinical information about oncology trials does not mitigate this internal struggle. Such trials aim for “the first-in-human study of a new investigational medicinal product […] establishing the optimal dose […] while determining the toxicity profile” or test for antitumor activity in a specific setting [16]. Although the risk–benefit ratio for participation in early-phase clinical trials has improved [41], the choice for patients to participate remains a delicate balance between a realistic chance of side effects and a slim chance of survival benefit [12, 24]. Entering such a crucial phase in life, patients’ decisions will therefore be built largely on core values [39], so-called proper values that can be recognized by their intersubjective character and their referral to everyday language (e.g., sincerity or love) [28, 39]. As decisions regarding early-phase clinical trial participation are strongly value laden and usually tragic in character, sharing core values and biases is ideally part of the patient-oncologist conversation [20].

Research has shown that the discussion of core values and preferences supports patient decision-making [8, 21]. Attention to patient values usually puts patient autonomy at the forefront (which results in full patient-centered decision-making [20]), can lead to understanding a patient’s “vision on the good life for themselves and their fellows” (which can result in well-informed and attuned advice by a physician [20, 28, 39]), and may lead to an equal sharing of values by patient and physician [7, 9, 21]. When core values were discussed, patients experienced a more active role during the decision-making process [9, 10, 21, 30]. However, despite the beneficial aspects of discussing core values, oncologists often neglect [13, 17] or only give a small role to discussing the values of patients [21, 33]. Given the complexity of contemporary early-phase clinical trials [19], the focus is often sharing technical information about the trials [3]. In addition, many patients find it difficult to discuss values with their oncologist [34]. It thus remains a challenge for both patients and trial oncologists to fully address patient values, although supportive, in the context of tragical circumstances in which decisions concerning participation in an early-phase clinical trial have to be made.

In a systematic review [36], we collected all patient values that have been previously reported in the international literature on participation in early-phase clinical trials: hope, trust in the health care system, quality or quantity of life, altruism, social adherence, autonomy, faith, perseverance, risk tolerance, and humanity. However, most previous studies on patients with advanced cancer, such as those by Catt et al. [4] and Walshe et al. [40], refer only to values non-intentionally (i.e., discussing coping mechanisms or reasons for participation), are not always (directly) linked to early-phase clinical trials, and, if so, they are usually focusing on patients after deciding whether or not to participate in such a trial. Moreover, most available studies work with pre-defined values, converted into survey questions.

To our knowledge, the values of adult patients with advanced cancer who are still reasonably fit and are confronted with the choice of participating in an early-phase clinical trial have not yet been inductively and longitudinally studied, especially not with a focus on the complex and tragic interplay of values within these patients. Such research is important, as early-phase clinical trials usually concern medication research with uncertain outcomes and probable side effects with a substantial impact on the life and well-being of the patient. This article identifies the core values that play a role in patients’ decision-making process about potential participation in early-phase clinical cancer trials.

## Methods

This article is part of a research project that aims to optimize shared decision-making processes for early-phase clinical trials in patient-oncologist communication in the near future [37]. Face-to-face, semi-structured serial interviews with patients with advanced cancer were considered the appropriate method for studying the values that are woven into patients’ common language about the good life [25, 28]. This method enables us to follow a patient’s complex decision-making process about participation in an early-phase clinical trial, which usually spans several weeks. Serial interviews were applied to generate more private and in-depth accounts of patients’ thoughts on the interplay of values in this particular phase of their lives [25].

## Recruitment and inclusion

**Fig. 1:**
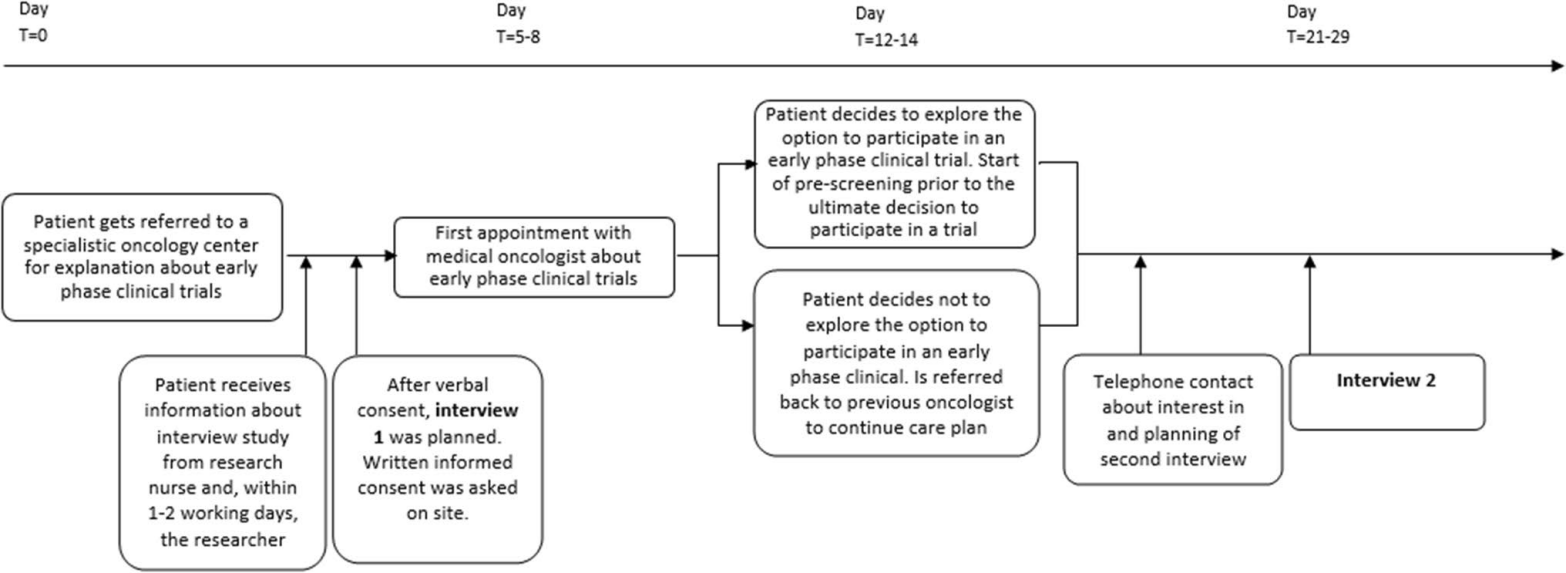
The process of recruitment, inclusion, and interviewing

Patient recruitment was organized in two Dutch specialized cancer centers, i.e., Erasmus MC Cancer Institute Rotterdam and the Netherlands Cancer Institute Amsterdam. All the approached patients had an initial consult planned on possible participation in an early-phase clinical trial. The process of recruitment, inclusion, and interviewing of patients is shown in Fig. 1. We started with convenience sampling and, after including 6 patients, switched to purposive sampling to add more diversity to the sample in terms of age (life phase) and gender [5], thereby considering that perceptions of core values may vary as a function of age, marital or cohabitation status, and upbringing [22]. Patient eligibility was determined based on the following inclusion criteria: the patient (1) had advanced cancer for which standard systemic therapy was not or no longer available, (2) was, in principle, eligible for first participation in an early-phase clinical trial, (3) was 18 years or older, and (4) was fluent in Dutch. The exclusion criterion was cognitive impairment. Patient interviews started in November 2018, and patients were included until October 2019, when saturation of the main issues occurred. Interviews were conducted at the patients’ preferred location, most often their home, and once at the patient’s workplace.

## Data collection

The interview guides for both interviews are based on a narrative medicine approach to invite patients to tell their illness story (Supplementary file 1). Illness, in itself, demands stories from patients to express what remains of the good life and to imbue it with meaning [14]. The first interview followed the structure of (a) talking about the present situation, including daily life, contact with medical services, and choices; (b) looking back on the period when a healthy life (abruptly) changed into a life with an illness; and (c) what interviewees considered important for the future. The second interview focused on the weeks following patients’ first appointment to discuss early-phase clinical trials, exploring which decisions were made regarding medical treatment and what these decisions meant for the patient’s outlook on life. Immediate transcript analysis from the first interview led to conversation topics and questions that could be further explored in the second interview [25].

The interview guide was tested with one patient for comprehensibility of the questions and overall coherence and then adjusted according to their feedback. During the interview process, small adjustments were made to the interview guide based on the first results from the analysis.

All interviews were performed by the third author (NS; trained junior researcher), digitally recorded, and transcribed verbatim by a research assistant. Field notes and memos were written about possible relevant events during the interviews. The raw data were uploaded into the CAQDAS program ATLAS.ti.

## Data analysis

Due to the explorative nature of this study, our qualitative analysis started with open coding [2, 6] delimited by Rescher’s distinction between “value objects that are being evaluated” (e.g., participating in the research), the “locus of value” (e.g., to help others obtain a cure), and the “underlying values” (e.g., altruism) [28]. To generate a cyclic analysis process in which previous interviews inform both the sampling and the interview guides, coding of the patient interviews commenced after the first interview. Two authors (N. S. and J. G.; please see Box 1 for more characteristics) independently coded the data from the first three series of interviews; the coding was shortly thereafter peer reviewed by J. H. Through comparison and discussion, incongruities in the coding were solved, and an initial coding scheme was set up. This initial coding scheme already contained a first categorization of codes under more abstract labels. In a second step, the coding scheme was further expanded and accentuated by NS, who coded the remaining ten series. During this process, the analysis shifted to axial coding and a greater emphasis on constant comparison to be able to identify solid categories and general themes and describe patterns and relationships [6]. In this step, three randomly selected series were independently coded and discussed by NS and JG using the coding scheme to check for reliability and validity of the analysis and to further stimulate creativity in coding [6].

To contribute to reliability and validity [1], NS used memos to record all important steps and choices in the analysis. Furthermore, the series of interviews and intervening analyses offered ample opportunities for formulating new questions and propositions that could be studied and contrasted in a later stage of the research. Serial interviewing enabled member checking within and between series. Finally, the eventual coding scheme was subjected to peer review by the research group.

**Table:** 

Box 1 Reflexivity statement
This article’s author group consists of researchers and clinician-researchers. The group varies in age, years of experience, and gender, and, in general, has a strong interest in improving shared decision-making and quality of care for patients with advanced cancer. All authors are born and raised in the Netherlands and received at least one (applied) university degree. The open coding enabled being true to the perspectives, voices, and words of the interviewees. The cyclic process of analysis made it possible to regularly member check the codes and categories from the developing coding scheme with new interviewees. Critical peer review came from the entire group of authors, which contains experts in cancer and palliative care, (health) communication sciences and anthropology, ethics, and health sciences Interviewer N. S. (F) is a junior researcher trained in doing qualitative research. She had no previous experience in the field of cancer research and no relationships with participants other than professional researcher-interviewee relationships. During the data collection and analysis, N. S. was supervised and supported by J. G. (M) and J. H. (M) who both have ample experience with qualitative research with seriously ill patients, ethics, cancer, and palliative care. Both supervisors did not have relationships with study participants

## Results

**Fig. 2:**
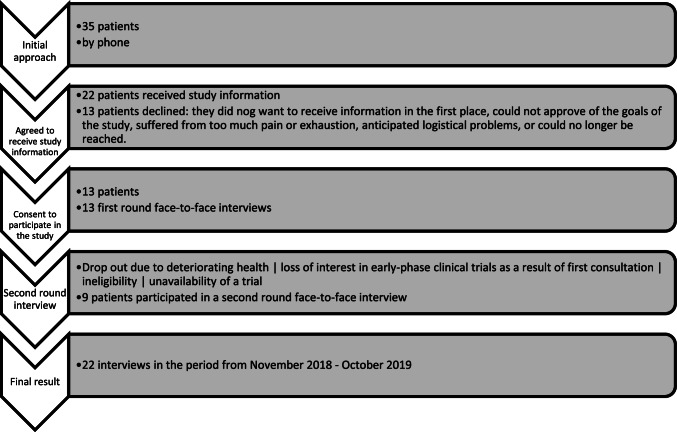
Flowchart of patient inclusion and interviewing

This study encompasses thirteen first-round in-depth interviews with patients, lasting 45–114 min, and nine follow-up interviews, lasting 38–79 min (Fig. 2). For the characteristics of the participants, please see Table 1.

**Table 1:** Characteristics of included patients with advanced cancer who participated in the interviews

Characteristics of patient participants	*N* = 13
Gender (male/female)	7/6
Age (years)	
Mean average (SD)	60.5 (11.0)
Range	32–69
Familial/home situation	
Living with partner	10
Living alone (divorced)	3 (2)
Presence of children/grandchildren	9/5
Religious affiliation (yes/no)	3/10
Cancer diagnosis	
Gastro-intestinal	5
Hepatobiliary/pancreatic	3
Gynecological	2
Lung	2
Small cell carcinoma, primary tumor unknown	1
First diagnosis (year)	
< 2014	1
2014–2017	9
> 2017	3
Participation in the early-phase clinical trial (information available after two interviews)	
Chose to participate/was able to participate	2
Unable to participate (not the required physical fitness, including lacking the correct mutation)	7
Chose not to participate	3
No information on decision	1

Whereas many people experience life as having an open future, this study’s participants stood face-to-face with the finiteness of life, although the exact prognosis was often unclear. The news, after rounds of treatment, that cancer had not reacted to treatment or even advanced, brought dying and death very close to home for the patients. In this context of having a severely limited future, pressing choices presented themselves. Whereas some patients tried to ignore their diagnosis as long as possible and wanted “to go back to normal” (P10), others reserved the remaining time for bucket list items and loved ones. A third group strived to look for treatment possibilities to prolong life or to stabilize the tumor. Participants considered, to a greater or lesser extent, six values central to their decision-making process whether to participate in an early-phase clinical trial.

### Acceptance creates room for reconsideration of values

Participants often spoke about continuous shifts in priorities as a consequence of an increasingly protesting and deserting body. During their time of illness, they had already learned to adapt to such malfunctions. Usually, acceptance came with adaptation. Some participants described the situation as accepting (a cruel) fate, even leading to habituating to the thought of passing away soon. In this process, activities lost importance, such as work, while other things gained importance, such as a focus on personal well-being or creating lasting memories with loved ones. In this context of being able to accept further decline, a choice for an early-phase clinical trial had to be made.

*P5: Accept that there are certain things you cannot do. Or can do less. Well, you do them tomorrow. The world can wait.*

*P8: When you are ill, a lot of things are no longer important. You only notice that when you’re ill.*

### Reconciliation with one’s fate

In general, participants tended to surrender to their fate. Their fate was beyond their span of control, an example of bad luck, or, for some participants, a result of their god’s mysterious ways. Such a surrender actually saves energy, which could then be applied to matters in daily life.

Those who thought their fate was somehow connected to their god’s providence found extra support in their religion to cope with their ill fate. Religion comforted them in providing an outlook on an afterlife and in giving them a god who would watch over their family when they were no longer there. Although participants could hold onto religion in decision-making processes (e.g., experiencing an obligation to withstand your fate as long as possible), some could not entirely reconcile their advanced cancer with their religious beliefs. However, with respect to participation in early-phase clinical trials, participants mentioned that these trials are rare opportunities that fate or providence had to offer and that one had to hope to be given the strength to make the best of this opportunity.

*P9: I simply took the blow. I try not to make a big issue out of things I can’t change. Because I think that is an unnecessary waste of energy.*

*P11: I’m guided [by God] and then I think: “Well, if this [early-phase clinical trial] crosses my path, it just has to be this way.”*

### Hope

Advanced cancer comes with receiving bad news on a regular basis. Some participants explained how they quickly shoved this bad news aside to remain open to new ways out of their difficulties and to be able to seize every opportunity that announces itself. “People keep living when having a little bit of hope.” (P2) Participants presumed that feeling down was not going to help. Neither was giving up. An optimistic outlook shows indomitability: an optimistic attitude is a prerequisite for a positive outcome. Although most participants admitted that a cure was unrealistic, they clung to acting out optimism by participating in early-phase clinical trials and new treatment options in general. Participants studied scientific reports, collected anecdotic evidence, and appreciated being referred to specialized centers. Other ways that were mentioned and explored were treatments abroad or alternative medicine.

*P11: If I get the opportunity, then I am going to take it. I do not think: “well, it won’t do anything”. So that is my positive attitude, so to say.*

*P3: I still feel really good. I have no issues. So, you take every offer with both hands. Even if it is an experiment. I believe you always have to have a bit of hope.*

### Autonomy: self-governance and relational autonomy

Having advanced cancer usually leads to bodily decay obstructing normal daily activities and causing a dependence on others for care or help with daily activities, mobility issues, or frequent hospital visits for treatment. Cancer invades both the body and social life. A chance to maintain or regain an autonomous and active life was part of the participants’ motivation to participate in an early-phase clinical trial. Such trials even support a sense of autonomy since patients are given the freedom to withdraw at any moment.

*P6: And then there’s the option to do nothing. […] I really disliked that option because you know your symptoms just get worse. […] And the pain. I would do everything, or perhaps try everything to at least explore the possibility of shrinking the tumor.*

*P2: She [the oncologist] swore to me that I could stop at any time. So if she calls and says, “I have this or that…”And we reply with, “Well, sorry, but we are not going to do that,” then we are just done [with participation in an early-phase clinical trial].*

In regard to decision-making, most participants indicated that they are the ones making the decision but that they will always consider the opinions of close ones, including close caregivers such as a general practitioner (GP). The decisions made by the patient would always affect others, and these effects had to be weighed as well. In return, participants hoped to be recognized and treated as persons first and patients second. This consideration implied that personal recognition by others had to be accompanied by a sensitivity to the ill person.

*P2: I am not alone [in deciding whether or not to participate]. I have a wife and children, and they all commiserate with me. I am not doing [the clinical trial] on my own.*

### Body preservation

For the interviewees, to keep the body moving meant both restraining physical deterioration and remaining in touch with life. Some participants were focused on optimizing the fitness of their bodies with food and exercise to enhance the chances for admittance to and successful trial participation. Other participants, in contrast, valued their body for its capacity to mediate pleasure by means of food, drinks, and/or other stimulants in times of misery. Participants remained ambivalent, as a patient mentioned the importance of dinner parties, but a loss of taste would not stop her from enrolling in an early-phase clinical trial because “life is bigger than certain things [dinner parties]” (P10).

### Altruism

The willingness to participate in an early-phase clinical trial benefits not only the participants themselves. A few participants hoped that future generations might benefit from the scientific results obtained in clinical trials in which they would participate.

*P10: Of course, there must be something at some point. I mean. Look, basically I am incurably ill. I know that, but I am not selfish in that I do not want to see if something is possible for other people. Right?*

### Patients in conflict

The interviewed patients occasionally expressed clear despair in which the abovementioned values played leading parts. Interviewees struggled with weighing the minimal chance for improvement and survival against the unknown risks and side effects. Stripped down to its essence it is their choice between potentially living longer and the risk of further invalidating their (quality of) life. Whereas the hope to live on, to live an autonomous life, needed to be performed in actions (i.e., their willingness to participate in a trial), the thoughts of further limiting social life (and shared decision-making), of having to live an ascetic life to keep the body ready for trial participation, or of the peace and energy that comes from accepting one’s fate continuously questioned these performative acts of hope. Within this area of tension, each interviewee made a personal assessment and usually showed some ability to accept and adapt to further decline in the end.

*P13: I will take that chemotherapy. But there are no guarantees that it will work. I am well aware of that. But I want, if possible, keep making plans for the future … I’m hoping that I can keep doing that.*

*P4: But you keep thinking: ‘Perhaps, in my lifetime, they will develop something else.’ You know. That’s why you are always saddled with this dilemma: ‘Am I done now? Or will I continue?’.*

## Discussion

This study aimed to qualitatively describe the core values that play a role in patients’ decision-making processes about participation in early-phase clinical trials in oncology before and after their first conversation with a trial oncologist. Patients generally see themselves confronted with several, sometimes conflicting, values, demanding careful appraisal against the background of tragic circumstances. Single values can therefore never be causally related to participation in early-phase clinical trials.

With the value *acceptance*, what sticks out is the positive association with adaptation, which has been described before [27]. When patients are able to take a stance of acceptance [42] toward their situation, the focus can shift to the problems ahead [18] and the accompanying relevant values. With respect to *hope* in this article, it seems fair to say that this value combines both hope and perseverance, as mentioned in Van Lent et al. [36]. Whereas perseverance appears in this review as unrealistic or “blind” hope, this study actually showed the flexibility and focus of patients who are willing to explore different paths to achieve their sometimes less realistic goals before downscaling to more realistic goals [26, 29]. These patients think that they have to perform their hope and that they actually have to do something to have a chance to reach their goals [32]. In contrast, within patients who only receive palliative care, this focus on self-direction and self-enhancement is no longer current. Although these patients still value independent thought and being able to choose their own actions, their focus shifts more toward relevant others (e.g., proxies, friends) [11].

In the work of Van Lent et al. [36] and Sulmasy et al. [32], being religiously faithful is presented as a justification for decision-making: God or the gods continue to mean well and will bless those who faithfully chose to participate in early-phase clinical trials. In the current study, however, participants present religious faith more as a comfortable backup for wherever your fate leads you: there is little to no talk about being chosen or becoming the miracle, while there is more talk about faith providing consolation that although your life recently became ill-fated, all will end well for you and your family. God or the gods appear to be more distant and sometimes even absent when confronted with both the harsh fate of advanced cancer and the more positive fate of getting an opportunity to participate in an early-phase clinical trial.

Although the body has been a relevant topic within cancer care for years (e.g., sexuality [23], taste [15]), *body preservation* did not surface as a patient value in the considerations for potential early-phase clinical trial participation before [36]. The (fitness of the) body is perceived as an important precondition for participation as well as success, likely also relating to a more general focus on physical fitness in Western society. However, investing in a fit body and further treatment in early-phase clinical trials can come with a tragic price: submitting the body to a trial can result in the permanent destruction of bodily senses that, at least partially, make life worthwhile.

It is, however, the interplay between different values as described in the results that stands out most. The immediate threat to life that our interviewees experienced, highly characteristic for this patient group in contrast with other not seriously ill patient groups [38], pushed them to at least explore the option for trial participation in hopes that their lives could be extended and improved. But while considering the option for trial participation, it was mainly the ongoing medicalization of life and of the body as well as the burden for family and friends which severely complicated this decision.

## Study limitations

For this article, serial interviewing was used to achieve depth in the interviews and not to study development or change within a person with regard to his or her values. Since the studied values are core values, a radical shift in values would not be expected. However, it would be interesting to study subtle shifts in persons’ appraisal. With life values usually being strongly influenced by age and upbringing, this study’s sample is insufficient to reach full saturation as both the oldest old and the young adults are missing, but all values depicted above were strongly represented throughout the raw data hence the reference to “saturation of the main issues.” Our data collection before and after the first consult with a medical oncologist implied that we could explore the role of values of those who were willing to consider participation in a trial in the decision-making process. However, four of the participants who actually experienced changes (e.g., were disappointed, rapid physical decline) refused to participate in a second interview.

## Clinical implications

Patients who can rightfully give words to their core values are usually more reflective, understand the role of values in their lives better, and are more capable of explaining them to others [39]. This article furthers our understanding of core values that play a role in the lives and decision-making of patients with advanced cancer who explore participation in early-phase clinical trials. This insight provides future patient-professional consultations on an early-phase clinical trial with the necessary profundity. This understanding also cultivates a sensitivity to what motivates patients’ emotions, thoughts, and decisions [35]. It supports mutual understanding and dialog through which patients are more likely to make decisions according to their “vision[s] on the good life for themselves and their fellows” [28, 31].

## Conclusion

Most patients who are initially interested in participating in an early-phase clinical trial usually rely on a delimited set of core values. These values do not necessarily have to be compatible with one another (e.g., performing hope to live on versus acceptance of illness and death, preserving the body for early-phase clinical trial participation versus enjoying the body for the time being), making tragic choices necessary. Giving space to these values in healthcare conversations can lead to a sensitivity for and dialog about the subtle and sometimes ambiguous thinking of patients concerning what constitutes a good life, and the role of participation in an early-phase clinical trial in this life.

## Supplementary Information

Below is the link to the electronic supplementary material.

Supplementary file1 (DOCX 21 KB)

## Data Availability

The (Dutch) data are available upon request.
