# Supplementary material for: Core values of patients with advanced cancer considering participation in an early-phase clinical trial: a qualitative study
Source: Support Care Cancer. 2022 Jun 8;30(9):7605–13. doi: 10.1007/s00520-022-07200-5 (PMC9385761; doi:10.1007/s00520-022-07200-5)
Supplement: Supplementary file 1 — Supplementary file1 (DOCX 21 KB) [file 520_2022_7200_MOESM1_ESM.docx]

**Supplementary file: TOPIC GUIDES Patient interviews**

*Interview 1*

| **Introduction** |
| --- |
| - The researcher introduces herself - Purpose of the interview and referring back to previous contact by telephone - Check whether the respondent has read and understood the information letter - Check if patient knows this is the first of two interviews - Interview takes about one to one and a half hours: “About your life before and after the diagnosis ‘advanced cancer’, what you deem important in life, future perspectives and making choices.” - Discuss confidentiality, audio recording, anonymization - Tired, emotional or uncomfortable? Please report this to me. We can then pause or even stop the interview. When I think that you are getting tired, I will ask you this as well. - Do you have any questions regarding this introduction/the study before we start? |
| **Individual history** |
| *What was central to your life before you got sick?*   - Description of a typical day/week - What did you think (experience/value) during the life before you got sick?   - Family/relatives/friends   - Work/community/leisure time   - …   *What was your perspective on your future?*   - What drove you?   *How did getting cancer affect the life you led?*   - Please explain how this changed your perspective on your future. |
| **Today** |
| *What is central to your life right now?*   - Description of a typical day/week;   - What do you (no longer do) do?   - What drives you?   - How do you value this life?     - Returning to the topic of family/relatives/friends and work/community/leisure time   *In what ways did you came into contact with the medical world?*   - What did/do you do with these contacts? - What do you consider important in these contacts? - You’re now facing (difficult) decisions:   - Which are these?   - How do you arrive at your choices?     - What considerations do you make in this regard?   - What do you think about your choices?   - Do you discuss the choices you make or have to make with others?     - If so, with whom? |
| **Future** |
| *Do you currently have any wishes? (things you want to experience or would like to do)?*   - How do you envision your (near) future? |
| **Closing** |
| The plan is to develop an online tool for patients with advanced cancer that will give the patient insight into his/her values ​​and more confidence in decisions made (whether or not to participate in an early phase clinical trial and/or palliative care )    Closure  -         Thank you for participating and for your openness  -         Possibility to talk to a social worker after the interview if there is a need for it  -         You can always contact us later if you have any questions or want to add something |

*Interview 2 – offer the possibility to do this interview via telephone*

| **Introduction** |
| --- |
| Thank you for welcoming me back for a second interview. Do you have any questions beforehand?  This interview lasts approximately one hour. This time it will be about your life after making a treatment choice and how you see the future. I will also return to a number of points that were discussed last time.  The conversation is confidential again. I would like to record it again. Anything you say that may refer to you will be made anonymous. If you no longer feel comfortable or tired during the interview, you can indicate this. We can then pause or stop. Do you have any questions? |
| **Today –** referring back to elements in the first interview |
| 1. *Can you tell what has happened in the past few weeks?*    1. How did you experience this period?    2. With what expectations did you enter the conversation with the oncologist?    3. Are there things that could have helped you during this time?       1. If so, what could have helped you?    4. You have made a (difficult) choice, how did you come to this choice?       1. What considerations have you made in this regard?       2. What do you think about the choice made?    5. Have you discussed the choice you had to make with others?       1. If so, with whom? If not, why not?       2. If so, how did this conversation take place? 2. *How are you doing now?/How do you see your life now?*    1. There are a number of things that came up last time that I would now like to go into in more detail. (three weeks ago you told […], how do you see this now?)    2. What has changed since you made a choice? |
| **Future -** referring back to elements in the first interview |
| - *Do you currently have any wishes? (things you want to experience or would like to do)*   - You will now have to make (difficult) decisions again:     - Which are these?   - What will your care process look like from now on?     - What does this mean to you?   - How do you envision your (near) future?     - How has this changed after you made your choice? |
| **Closing** |
| The plan is to develop an online tool for patients with advanced cancer that will give the patient insight into his/her values ​​and more confidence in decisions made (whether or not to participate in an early phase clinical trial and/or palliative care )    Closure  -         Thank you for participating and for your openness  -         Possibility to talk to a social worker after the interview if there is a need for it  -         You can always contact us later if you have any questions or want to add something |
